# Identification of barriers and facilitators in nationwide implementation of standardized structured reporting in pathology: a mixed method study

**DOI:** 10.1007/s00428-019-02609-6

**Published:** 2019-07-03

**Authors:** J. E. M. Swillens, C. E. Sluijter, L. I. H. Overbeek, I. D. Nagtegaal, R. P. M. G. Hermens

**Affiliations:** 1 Radboud Institute for Health Sciences (RIHS), Scientific Institute for Quality of Healthcare (IQ Healthcare), Radboud University Medical Center, Geert Grooteplein 21, POB 9101, 6500 HB Nijmegen, The Netherlands; 2 PALGA Foundation, Randhoeve 225A, 3995 GA Houten, The Netherlands; 3 Radboud Institute for Molecular Life Sciences (RIMLS), Department of Pathology, Radboud University Medical Center, Geert Grooteplein-Zuid 10, POB 9101, 6500 HB Nijmegen, The Netherlands

**Keywords:** Pathology, Standardized structured reporting, Quality, Implementation, Guideline adherence

## Abstract

Standardized structured reporting (SSR) enables high-quality pathology reporting, but implementing SSR is slow. The objective of this study is to identify both barriers and facilitators that pathologists encounter in SSR, in order to develop tailored implementation tools to increase SSR usage. We used a mixed method design: a focus group interview helped to identify barriers and facilitators in SSR. The findings were classified into the following domains: innovation, individual professional, social setting, organization, and economic and political context. We used a web-based survey among Dutch pathologists to quantify the findings. Ten pathologists participated in the focus group interview, and 97 pathologists completed the survey. The results of both showed that pathologists perceive barriers related to SSR itself. Particularly its incompatibility caused lack of nuance (73%, *n* = 97) in the standardized structured pathology report. Regarding the individual professional, knowledge about available SSR-templates was lacking (28%, *n* = 97), and only 44% (*n* = 94) of the respondents agreed that using SSR facilitates the most accurate diagnosis. Related to social setting, support from the multidisciplinary team members was lacking (45%, *n* = 94). At organization level, SSR leads to extra work (52%, *n* = 94) because of its incompatibility with other information systems (38%, *n* = 93). Main facilitators of SSR were incorporation of speech recognition (54%, *n* = 94) and improvement in communication during multidisciplinary team meetings (69%, *n* = 94). Both barriers and facilitators existed in various domains. These factors can be used to develop implementation tools to encourage SSR usage.

**Electronic supplementary material:**

The online version of this article (10.1007/s00428-019-02609-6) contains supplementary material, which is available to authorized users.

## Introduction

Discussion of each patient in multidisciplinary team (MDT) meetings is fundamental to optimal treatment decisions [1]. The complexity of modern multidisciplinary oncological diagnostics and treatment warrants timely, complete, adequate, and accurate exchange of information based on pathology reports. Optimization of pathology reports may save time and money [2].

The use of standardized structured reporting (SSR) in pathology instead of the traditional narrative, free-text reporting (NR) improves exchange of diagnostic and treatment information [3]. As a consequence of improved communication, MDT-members can take more appropriate treatment decisions, which leads to improved patient outcomes [4–8]. Multiple national and international guidelines, including those of the College of American Pathologists (CAP) [9], the Royal College of Pathologists (RCP) [10], the Dutch guidelines [11], and International Collaboration on Cancer Reporting (ICCR) [12], therefore recommend SSR. SSR includes an electronic reporting template with standardized reporting language, multiple-choice answering of parameters, and automated conclusion generation, which leads to well-structured overviews of the essential parameters of specific cases [13]. SSR is also important to secondary users including tumor registry organizations, health planners, epidemiologists, and others involved in healthcare quality-improvement activities and research [13–18].

The use of SSR in pathology varies widely across laboratories and tumor types [19–23]. In the Netherlands, we introduced SSR in 2009. After 9 years of implementation, SSR is now used in 86% of the colorectal cancer resections and 81% of the breast cancer resections. Over the years, SSR was introduced for another 20 cancer types, resulting in 27 different SSR-templates in total. The actual use of the SSR-templates varied from 8 to 86% (median 54%) in 2017 [24]. The wide range of actual SSR use in the Netherlands is comparable to other countries [19–23]. Therefore, despite some success with passive implementation, there is considerable room for improvement to the extent and speed of implementing SSR in pathology.

The adoption of innovations such as SSR is laborious, particularly when innovation requires people to change their daily habits [25]. Offering implementation tools facilitates adoption of innovations among medical professionals. Implementation tools are most effective when they take into account factors that impede or facilitate use of the innovation [26–28]. Therefore, the aim of this study was to explore barriers and facilitators in SSR-implementation among pathologists in a nationwide setting.

## Methods

### Study design

We used a mixed method design. We used a qualitative focus group interview to identify barriers and facilitators that Dutch pathologists encountered in SSR. We used a web-based survey to quantify the findings: to identify possible determinants and to assess the importance of the barriers and facilitators.

### Setting

PALGA, the nationwide network and registry of histo- and cytopathology in the Netherlands, was established in 1971 and achieved nationwide coverage in 1991, which means that all 46 Dutch pathology laboratories are connected to the PALGA network. The PALGA database contains the reports of all pathology tests in the Netherlands. This database is used for supporting patient care, for evaluating and monitoring the population screening programs, and for scientific research purposes [29]. More detailed information about PALGA can be found in additional file 1.

PALGA introduced SSR by providing their nationally available SSR-templates [30]. The Dutch SSR-templates are electronic reporting templates based on national evidence-based guidelines and WHO guidelines [31], with standardized reporting language, multiple-choice answering of parameters, and automated conclusion generation, generating a standardized structured pathology report. At the time of data collection, 21 nationwide SSR-templates were available. The use of the colon biopsy SSR template is mandatory for registering data for the national screening program for bowel cancer [24]. The PALGA SSR-templates are considered level 6, the highest level of the Spectrum of Cancer Pathology Reporting [19]. In additional file 2, the PALGA SSR-template of breast cancer biopsy is shown. To accurately refer to this level 6 type of reporting, we chose to use the term “standardized structured reporting” (SSR).

Other important pathology associations are the Dutch Society of Pathology (NVVP) and the Dutch Society of Pathology Residents (LPAV). Both pathologists and residents are members of the NVVP, whereas the LPAV only allows residents. At the time of data collection, 395 pathologists [32] and 105 residents were active in the Netherlands [33].

### Participants

#### Focus group interview

One of our researchers (CS) purposively e-mailed 30 pathologists. We aimed to include pathologists from university and non-university hospitals with different lengths of experience, as well as the range of opinion leaders, middle majority, and stragglers regarding SSR. The participating pathologists filled in a short questionnaire consisting of questions about the pathologists’ characteristics, clinical setting, and SSR usage. They also signed informed consent forms for recording the focus group interview and for anonymously analyzing the data obtained.

#### Survey

We invited and reminded pathologists to complete the survey via the PALGA network. The PALGA liaisons distributed the survey to their pathologist colleagues. Further, a call to complete the survey was posted on the PALGA webpage (www.palga.nl/nieuws), on the PALGA LinkedIn page, and in the newsletters from the Dutch Society of Pathology Residents and the Dutch Society of Pathology for 4 consecutive weeks. The introduction of the questionnaire informed the respondents about the research question and the anonymous data analysis. Completing the questionnaire took 10 to 15 min.

### Instrument development and data collection

#### Focus group interview

We used the domains of the theoretical models of Flottorp et al. [28] and Grol and Wensing [27] to develop an interview guide, with which we structured the focus group interview. The focus group interview took place at the annual PALGA day. An independent chairman, former head of the Radboudumc pathology department, supervised the focus group. The researcher (CS) started the focus group interview with an explanation of the research question. Then participants introduced themselves by explaining their sub-specialism within pathology, if applicable, and their experience with SSR. Next, the chairman asked about existing barriers and facilitators in the various domains. At the end, participants had time for final remarks. The interview was audio-taped and transcribed verbatim for content analysis with Atlas.ti (version 7.5.15, Atlas.ti Scientific Software Development; Berlin, Germany).

#### Survey

The survey was developed on the basis of the barriers and facilitators in SSR-implementation identified in the focus group interview, and some factors were added from literature as well [27, 28]. We used LimeSurvey (version 2.06+) to develop a web-based survey. The online survey did not accept unanswered questions, but the respondent could end the survey at any given time. The first part of the survey contained questions about the pathologists’ characteristics and their clinical settings. The second part of the survey consisted of seven theses and one open-ended question about SSR usage, as well as 61 theses to quantify the barriers and facilitators in SSR-implementation (both the use of SSR-templates and standardized structured pathology reports). These theses were classified in five main domains. To prevent repeated answers for all items, some theses were formulated in reverse. The theses were scored on a five-point Likert scale (strongly disagree (0) to strongly agree (4)), and when relevant, “Some do, others do not” (2) was included. A high mean score implies a facilitator, whereas a low score implies a barrier. At the end of the survey, participants could state their preferences, name additional barriers and facilitators, and suggest improvements for SSR-implementation.

### Outcome measures

The primary outcome measure was the set of identified barriers and facilitators for implementing SSR. The secondary outcome measures were determinants associated with barriers and facilitators.

### Data analyses

#### Focus group interview

Two researchers (CS and LO) independently extracted barriers and facilitators in SSR-implementation from the transcribed interview. The factors identified were allocated to the five domains. The two researchers discussed any discrepancies until they achieved consensus.

#### Survey

We used IBM SPSS Statistics 24 to analyze the survey results. We included questionnaires with at least 50% of the answers completed [34]. We used descriptive statistics to analyze participant characteristics and to quantify agreement with the theses in the survey. We categorized the agreement scores as “strongly agree,” “agree,” and “strongly disagree.” The category “strongly disagree” includes the answer option “Some do, some don’t.” Because this was a self-developed survey, we used psychometric properties (Cronbach’s *α*) and component analyses to confirm the reliability of the questionnaire. We turned negative items around to calculate Cronbach’s *α* and mean scores. We found high Cronbach’s *α*’s in all domains or sub-domains; credibility (.85), feasibility (.89), compatibility (.76), knowledge and skills (.95), attitude (.75), social setting (.80), and organizational factors (.74). We deemed the questionnaire reliable by these results. We used conventional content analysis to analyze the qualitative comments in the survey [35].

In analyzing possible determinants at domain level, we used Cronbach’s *α* to check the consistency of the domain and calculate the mean score for each domain. Further, we employed multivariable linear regression analyses to investigate the existence of possible determinants of barriers and facilitators identified [36]. Based on literature and differences in agreement with the barriers and facilitators in SSR-implementation noted in the focus group interview, we investigated various determinants: pathologist and hospital characteristics, such as age, gender, career stage, number of colleagues within the pathology department; and employment at a university or non-university hospital. We first examined the associations between one determinant and all dependent variables (i.e., domains of influencing factors) with univariate linear regression analyses. We included only those determinants associated with the dependent variables (two-sided *p* ≤ 0.20) in the multivariable linear regression analyses.

## Results

### Study population

Ten pathologists participated in the focus group interview—five men and five women—with an average of 14.9 years of experience (3–30 years). Three pathologists worked in a university hospital and seven in a non-university hospital.

The survey yielded 119 responses, of which 97 had at least the minimum number of answers required (82%). Table 1 outlines the participant characteristics. The mean number of years of experience as a pathologist was 11.0, ranging from 0.2 to 30.0 years.

**Table 1:** General characteristics of survey respondents

Mean age (SD)		43.7 (10.8)	
Mean years of experience (SD)		11.0 (8.7)	
Mean amount of colleagues (SD)		15.8 (9.4)	
		Number of respondents	Percentage
Gender	Male	43	44.3
	Female	54	55.7
Sub-specialization^a^	Yes	56	57.7
	No	41	42.3
Personnel type	Pathologist	76	78.4
	Resident	21	21.6
Type of hospital	University hospital	35	36.1
	Non-university hospital	62	63.9
Use of SSR	Frequent	80	82.5
	Sporadic	13	13.4
	Never	4	4.1

Total number of respondents = 97

*SD*, standard deviation; *SSR*, standardized structured reporting

^a^Specialization within pathology

### Use of SSR

Of the survey respondents, 82% (*n* = 97) frequently used SSR. On average, 61% of the SSR-templates were used. The main reasons for not using SSR-templates were non-availability for specific tumors, the teaching setting of the laboratory, lack of awareness of available templates, lack of nuances in SSR, and the perceived extra time necessary for SSR. Most respondents (68%; *n* = 93) preferred SSR, 18% (*n* = 93) preferred NR, 4% (*n* = 93) preferred a local template, and 10% (*n* = 93) had no opinion. Furthermore, 28% (*n* = 97) lacked knowledge about the available SSR-templates. Most pathologists received information about updates of SSR-templates via their PALGA liaison (56%; *n* = 97).

### Barriers and facilitators

**Fig. 1:**
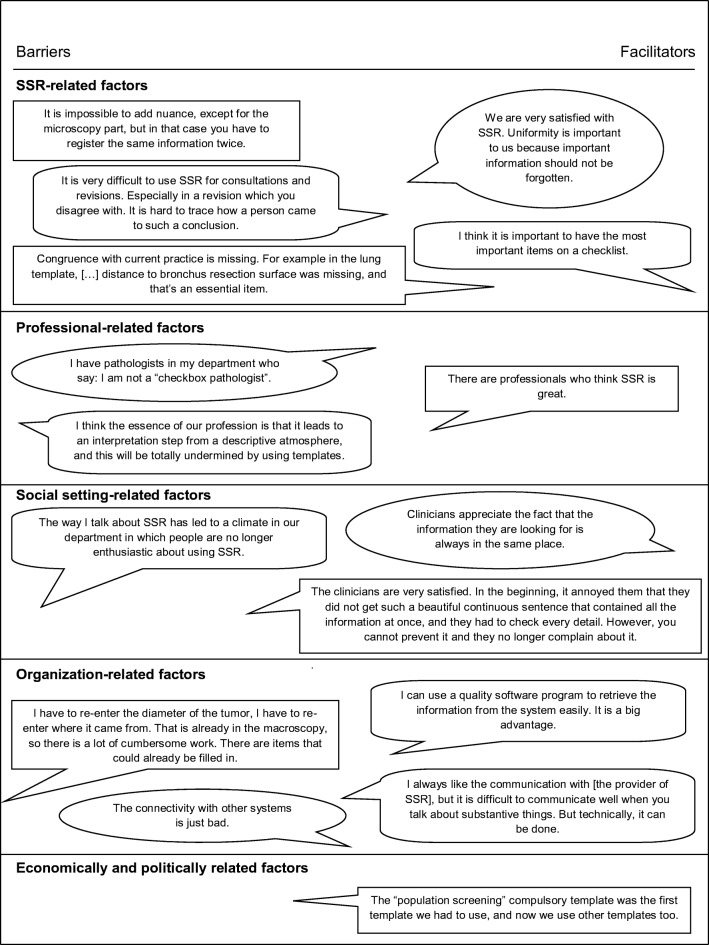
Pathologists’ illustrative quotes about barriers and facilitators in standardized structured reporting in the Netherlands. SSR, standardized structured reporting

**Fig. 2:**
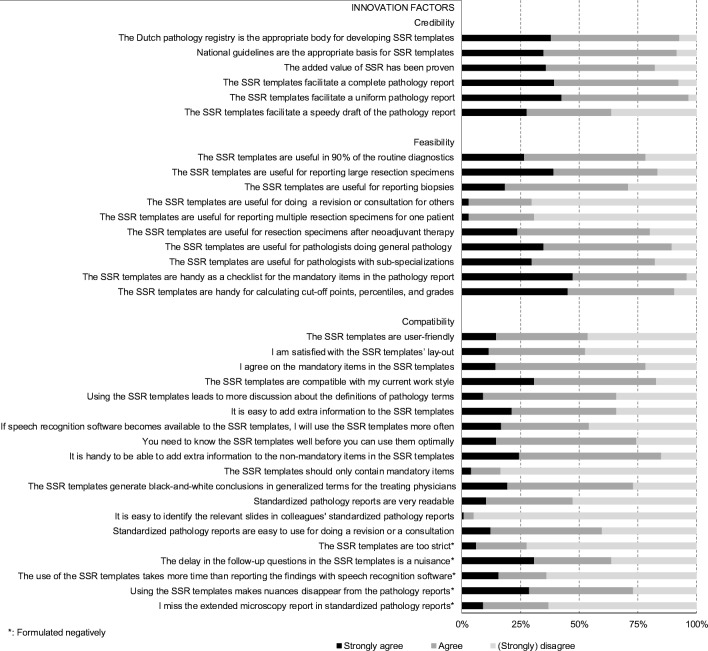
Answers to theses about the innovation factors of SSR. SSR, standardized structured reporting

**Fig. 3:**
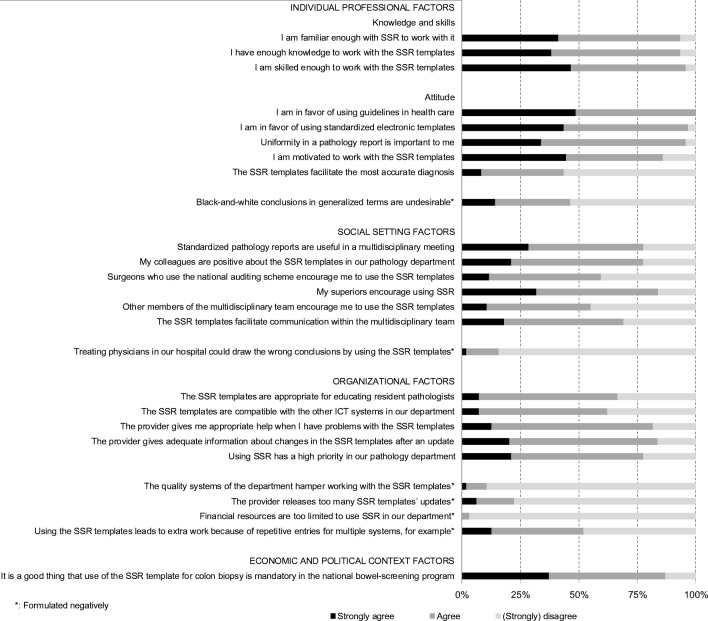
Answers to theses about the individual professional factors, social setting factors, organizational factors, and economic and political context factors. SSR, standardized structured reporting

Thirty-two barriers and 29 facilitators in SSR-implementation were identified in the focus group interview. Thirteen factors were considered to be both barriers and facilitators. Figure 1 illustrates the barriers and facilitators in SSR-implementation noted in the focus group by domain. All influencing factors were used in the web-based survey. Figures 2 and 3 show the survey answers. The most remarkable barriers and facilitators in SSR-implementation are discussed by domain.

### Innovation: SSR

Pathologists mentioned influencing factors related to the credibility, feasibility, and compatibility of SSR (Fig. 2). First, SSR-templates did not facilitate a speedy draft of the pathology report (36%; *n* = 94). However, 82% (*n* = 97) of the respondents acknowledged the added value of SSR, since it facilitates a uniform (97%; *n* = 94) and complete (93%; *n* = 94) pathology report.

Second, feasibility of SSR depended on the type of specimen. SSR-templates are inadequate for the reporting of multiple specimens per patient (69%; *n* = 97), for reporting revisions or consultations (70%; *n* = 97), and for reporting biopsies (29%; *n* = 97). However, SSR-templates are feasible for 90% of the routine diagnostics (78%; *n* = 97), including large resections (84%; *n* = 97), and, in contrast to the focus group findings, even after neoadjuvant therapy (80%; *n* = 97). SSR could be used by all pathologists, but are more attractive to pathologists without a sub-specialization (90%; *n* = 97). Furthermore, SSR-templates are supportive tools, either a checklist (96%; *n* = 97) or a calculator for cut-off points (91%; *n* = 97).

Third, SSR provided the respondents with difficulties in identifying relevant slides in colleagues’ standardized structured pathology reports (95%; *n* = 97), lacked nuances in these pathology reports (73%; *n* = 97), and showed poor report readability (53%; *n* = 95). In addition, the faltering of follow-up questions when clicking was undesirable (64%; *n* = 94). However, 83% (*n* = 94) stated that SSR were compatible with current practices. Specifically, SSR should not include only mandatory items (84%; *n* = 97) because it was helpful to be able to add extra information to non-mandatory items (85%; *n* = 94). The pathologists also agreed with these mandatory items (78%; *n* = 97). If speech recognition was added to the SSR, 54% (*n* = 94) would use it more often.

### Individual professional

Figure 3 reveals the items in the domains of the individual professional, social setting, organization, and economic and political context. The respondents were familiar with SSR (94%; *n* = 94) and had sufficient knowledge (94%; *n* = 94) and skills (96%; *n* = 94) for using SSR. With regard to attitude, only 44% (*n* = 94) agreed that the use of SSR facilitated the most accurate diagnosis, and 46% (*n* = 97) agreed that black-and-white conclusions in generalized terms are undesirable. However, all respondents were in favor of guidelines, and 97% (*n* = 94) were also in favor of SSR and motivated to use them (86%; *n* = 94). Uniformity of the pathology report is important (86%; *n* = 94).

### Social setting

Forty-five percent (*n* = 94) of the survey respondents lacked support from MDT-members to use SSR. However, SSR facilitated communication within the multidisciplinary team (69%; *n* = 94). The focus group participants mentioned that clinicians receiving the standardized structured pathology report had to become familiar with this new way of reporting and did not universally agree with the SSR format. The survey respondents stated that superiors (84%; *n* = 94) and colleagues in the pathology department (78%; *n* = 94) encouraged SSR and considered it useful in MDT-meetings (78%; *n* = 94).

### Organizational factors

According to 33% of the respondents (*n* = 93), SSR was considered inappropriate for the education of residents. Both the focus group interview and survey respondents (52%; *n* = 94) said that using SSR leads to more work because data must be entered into multiple systems. In addition, SSR was incompatible with other departmental software systems (38%; *n* = 93). However, the quality systems of the laboratories did not affect the use of SSR (89%; *n* = 93). Sufficient financial resources were available to the laboratories to use SSR (97%; *n* = 93), and using SSR also had high priority in the respondents’ pathology departments (78%; *n* = 94). Furthermore, PALGA delivered appropriate information about changes after a SSR update and development of new SSR (84%; *n* = 93).

### Economic and political context

The participants did not name any barriers in the economic and political context during the focus group interview. The respondents (87%; *n* = 94) reported that a mandatory SSR-template, such as for the national screening program for bowel cancer, is a facilitator.

### Improvements for SSR

Proposed improvements for SSR entailed improving the readability, nuance, and adaptability of the generated report; improving compatibility with other hospital IT systems; reducing or increasing the mandatory minimal dataset; and incorporating speech recognition software for both the main content and additional text fields.

### Determinants of the barriers and facilitators in SSR

The mean scores for all domains and sub-domains were above 2.1 (Likert scale 0–4) (Table 2). Univariate determinant analyses resulted in multiple determinants at professional and hospital level for (1) credibility, (2) knowledge and skills, and (3) social setting. These were gender and years of experience (*p* = .191 and *p* = .191), career stage and type of hospital (*p* = .034 and *p* = .090), and being a specialist or not and type of hospital (*p* = .138 and *p* = .182), respectively. Additionally, determinants for compatibility and attitude were found. These were being a specialist or not (*p* = .056) and gender (*p* = .151), respectively. After multivariable analyses (Table 2), career stage remained a possible determinant for knowledge and skills of SSR (mean scores: residents, 2.98; pathologists, 3.38; *p* = .034); pathologists’ career stage had an influence on the knowledge and skills of pathologists to work with SSR.

**Table 2:** Determinant analyses

Domain		Number of items	Number of respondents	Cronbach’s alpha	Mean score (SD)	Determinants	*p*
1	Innovation: SSR						
	Credibility	6	94	.85	3.06 (.78)		
	Feasibility	10	97	.89	2.66 (.77)		
	Compatibility	19	94	.76	2.15 (.51)		
2	Individual professional						
	Knowledge and skills	3	94	.95	3.30 (.75)	Career stage	.034
	Attitude	6	94	.75	2.84 (.65)		
4	Social setting	7	94	.80	2.60 (.78)		
5	Organizational factors	9	93	.74	2.62 (.61)		
6	Economic and political context	1	94	.	3.05 (1.10)		

*SD*, standard deviation; *SSR*, standardized structured reporting

## Discussion

This study has uncovered several important factors in the implementation of SSR in all domains of the theoretical models of Flottorp et al. [28] and Grol and Wensing [27]. The main barriers were usually related to infeasibility and incompatibility of SSR with pathologists’ current practices. Other important barriers were lack of knowledge about available SSR-templates, disbelief about the capability to deliver the most accurate diagnosis, disencouragement of SSR usage by MDT-members, and repetitive entries for multiple systems. The main facilitators related to multiple domains and sub-domains as well. The most important facilitators were incorporation of speech recognition in SSR, SSR uniformity of pathology reports, having superiors encouraging SSR, improved communication during MDT-meetings, and the mandatory SSR use for registering colon biopsy in the national screening program for bowel cancer. The only determinant of SSR-implementation appeared to be career stage. This is a minor contrast to earlier studies, that identified age rather than career stage as a determinant of implementation [14, 15, 20, 37]. The influencing factors of the innovation (SSR) and the individual professional factors are most relevant in settings with a high level SSR (level 6 of the Spectrum of Cancer Pathology Reporting [19] or higher). Moreover, the influencing social setting factors, both in the laboratory and MDT-setting, are relevant in countries that have multidisciplinary health care organization. Full use of the influencing organizational and political and economic context factors can be made in the setting of national or regional organizations that coordinate SSR.

Pathologists’ reluctance to implement SSR is visible in multiple studies [22, 23, 37–40]. Our study confirms previous findings that incompatibility with pathologists’ current practices is an important barrier [39]. Interestingly, most barriers related to the use of the standardized structured pathology report rather than the SSR-template. Before improving the readability, nuance, and adaptability of the standardized structured pathology report as the pathologists in this study and Ellis and Srigley [13] proposed, other MDT-members’ perceptions of compatibility of the standardized structured pathology report should been taken into account. The study by Lankshear et al. [38] shows a correlation between clinicians’ satisfaction and the information level along with easy information retrieval needed for patient management. Therefore, future research should aim at the clinicians’ perceptions of the Dutch SSR system to determine whether MDT-members perceive barriers in the standardized structured pathology report. If MDT-members do not face barriers relating to incompatibility of the standardized structured pathology report, they may play an important role in encouraging SSR use among pathologists [28].

An important barrier of SSR is an increased workload due to multiple incompatible systems, which require additional input of data [22, 23, 38]. Hassell and colleagues [23] propose adapting the pathologist workflow and reporting format as a solution. Additional research, such as an explorative focus group interview with the PALGA liaisons, is needed to clarify the barriers to multiple system use.

Multiple studies have published findings regarding the improved overall completeness of a pathology report when SSR (defined by Ellis and Srigley [13] as level 3 or higher) instead of NR, is used to report pathology evaluation [14, 15, 17, 19, 20, 22, 41–46]. In this study, we assume that the use of SSR facilitates a complete and uniform report. Future research should explore whether the end-users of the pathology SSR, indeed, classify the standardized structured pathology reports as complete and adequate for treatment decision making.

Incorporation of the findings from the focus group interview and survey in the implementation strategy of SSR should be used to develop a tailored implementation strategy. First, communication about availability of SSR for specific tumors should be improved, by providing clear guidelines to the PALGA liaison’s in the laboratories.

Second, to improve the feasibility and compatibility of SSR for pathologists, there should be a focus on training; for example, by e-learning or instruction videos [28]. The positive effects of such implementation training sessions about SSR usage has been observed in multiple studies [17, 22, 47, 48]. Most importantly, the use of SSR-templates in cases of multiple resection specimens should be discussed. Because residents have less knowledge and skills to work with SSR, the training program could be adapted to career stage.

Third, to overcome the negative attitudes of pathologists not using SSR, Srigley et al. [19] successfully used multiple funding sources as incentives for increasing the use of SSR in Ontario. Hassell et al. [23] suggest financial benefits, for example, grant programs, to encourage SSR use among pathologists. This study shows that mandatory registration was an important facilitator. Therefore, the incentive of additional funding of SSR could be discussed among secondary users of SSR who benefit from SSR.

Fourth, another important barrier to address is the use of multiple software programs in laboratories, which interrupts the workflow of pathologists and requires more time due to repetitive data entry. As Hassell and colleagues propose, the different software programs used to complete the standardized structured pathology report could be more integrated to facilitate a more efficient workflow and registry process. Speech recognition could be incorporated in SSR-templates for both the main content and additional text fields. Further, clinical data and macroscopy findings may be transferred automatically in the SSR-template. In this way, data is registered only once at the source and can be used multiple times.

### Strengths and limitations

The use of both qualitative and quantitative methods is a strength in our study. We selected the participants of the focus group with care to minimize the selection bias. This resulted in a diverse group including opinion leaders, middle majority, and stragglers. Our intention was to gain a view as broad as possible of any influencing factors. Additionally, we conducted a web-based survey among all pathologists in the Netherlands to quantify the barriers and facilitators in SSR named in the focus group. This enabled us to determine the most important barriers and facilitators.

There are some limitations as well. Due to the open invitation to the web-based questionnaire, we could not calculate the response rate. With 97 questionnaires, we may have some selection bias in our web-based survey. However, the main characteristics (gender, age, personnel type, and type of hospital) of the respondents represent the Dutch population of pathologists and pathology residents. In addition, the results showed that the respondent preferences were diverse, and even when pathologists preferred SSR, they still mentioned barriers. Moreover, only pathologists were included in this study. The pathology report readers, who are clinicians such as oncologists, surgeons, and radiologists, should be included to obtain a complete overview of the possible influencing factors. These clinicians are part of the MDT that uses the standardized structured pathology reports to take treatment decisions.

In conclusion, this study identified barriers to and facilitators for implementing SSR among pathologists in the Netherlands. Implementation tools based on these factors could increase the extent and speed of SSR-implementation. We would recommend investigating barriers to and facilitators for SSR-implementation among clinicians receiving pathology reports such as oncologists, surgeons, and radiologists to further increase the use of SSR.

## Electronic supplementary material

Additional file 1 General information about the PALGA foundation (PDF 7 kb)

Additional file 2 Screenshots of the Dutch PALGA SSR-template of breast cancer biopsy (*translated from Dutch to English*) (PDF 448 kb)
