# Supplementary material for: Identification of barriers and facilitators in nationwide implementation of standardized structured reporting in pathology: a mixed method study
Source: Virchows Arch. 2019 Jul 3;475(5):551–61. doi: 10.1007/s00428-019-02609-6 (PMC6861434; doi:10.1007/s00428-019-02609-6)
Supplement: Supplementary file 1 — General information about the PALGA foundation (PDF 7 kb) [file 428_2019_2609_MOESM1_ESM.pdf]

Additional file 1 General information about the PALGA foundation

Identification of barriers and facilitators in nationwide implementation of standardized structured reporting in pathology: a mixed method study

Virchows Archiv

J.E.M. Swillens, MSc, C.E. Sluijter, MSc, L.I.H. Overbeek, PhD, Prof I.D. Nagtegaal, MD, PhD, R.P.M.G Hermens, PhD

J.E.M. Swillens
Radboud Institute for Health Sciences (RIHS), Scientific Institute for Quality of Healthcare (IQ Healthcare), Radboud University Medical Centre, Geert Grooteplein 21, POB 9101, 6500 HB, Nijmegen, The Netherlands
Julie.Swillens@radboudumc.nl

The PALGA Foundation was established in 1971. The purpose of the nationwide network and registry of histo- and cytopathology in the Netherlands (PALGA Foundation) is to promote communication and the provision of information within and between pathology laboratories, and to make the resultant knowledge available to health care.

The PALGA network includes every pathology laboratory in the Netherlands. PALGA contains the results of every single pathology test: malignancies, benign disorders, as well as the results of tests in which no abnormalities were found. The PALGA network enables the best possible use to be made of data that are recorded in the laboratory.

The data in the central system forms the basis for the national cancer registry. It is indispensable for the evaluation and monitoring of population screening programs, it supports patient care, and it can be used for the purposes of scientific research. PALGA facilitates the fast, effective and economical use of pathology data from all Dutch pathology laboratories.

PALGA is subsidized by the Dutch Ministry of Health, Welfare and Sport.

Public pathology database

In the Netherlands, all pathology results are digitally archived in the nationwide network and registry of histo- and cytopathology in the Netherlands (PALGA). This means that all 46 pathology laboratories serving the pathology needs of every hospital in the Netherlands are connected to the national infrastructure. Each year, the pathology laboratories generate 2 million new test results in the areas of cytology (and population cytology), histology and autopsies. At the end of 2017, a total of more than 72 million pathology results from over 12 million patients had been stored. For many years, pathologists have worked towards the construction of this world-class archive, which is unique both in terms of its size and of the nationwide cooperation involved.

Additional information on the PALGA Foundation can be found on: https://www.palga.nl/en/
