# Supplementary material for: Identification of barriers and facilitators in nationwide implementation of standardized structured reporting in pathology: a mixed method study
Source: Virchows Arch. 2019 Jul 3;475(5):551–61. doi: 10.1007/s00428-019-02609-6 (PMC6861434; doi:10.1007/s00428-019-02609-6)
Supplement: Supplementary file 2 — Screenshots of the Dutch PALGA SSR-template of breast cancer biopsy (translated from Dutch to English) (PDF 448 kb) [file 428_2019_2609_MOESM2_ESM.pdf]

Additional file 2 Screenshots of the Dutch PALGA SSR-template of breast cancer biopsy (*translated from Dutch to English*)

Identification of barriers and facilitators in nationwide implementation of standardized structured reporting in pathology: a mixed method study

Virchows Archiv

J.E.M. Swillens, MSc, C.E. Sluijter, MSc, L.I.H. Overbeek, PhD, Prof I.D. Nagtegaal, MD, PhD, R.P.M.G Hermens, PhD

J.E.M. Swillens
Radboud Institute for Health Sciences (RIHS), Scientific Institute for Quality of Healthcare (IQ Healthcare), Radboud University Medical Centre, Geert Grooteplein 21, POB 9101, 6500 HB, Nijmegen, The Netherlands Julie.Swillens@radboudumc.nl

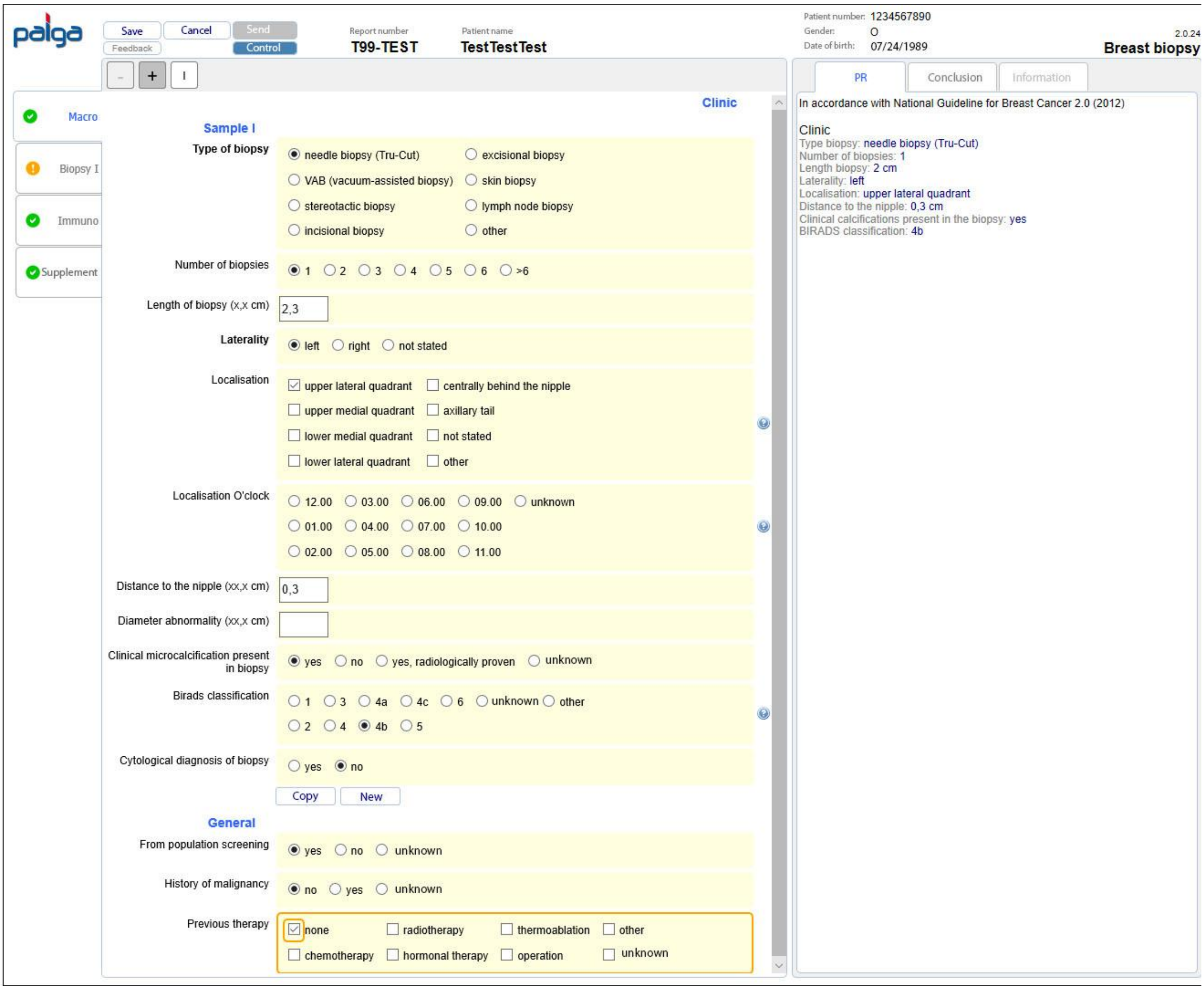

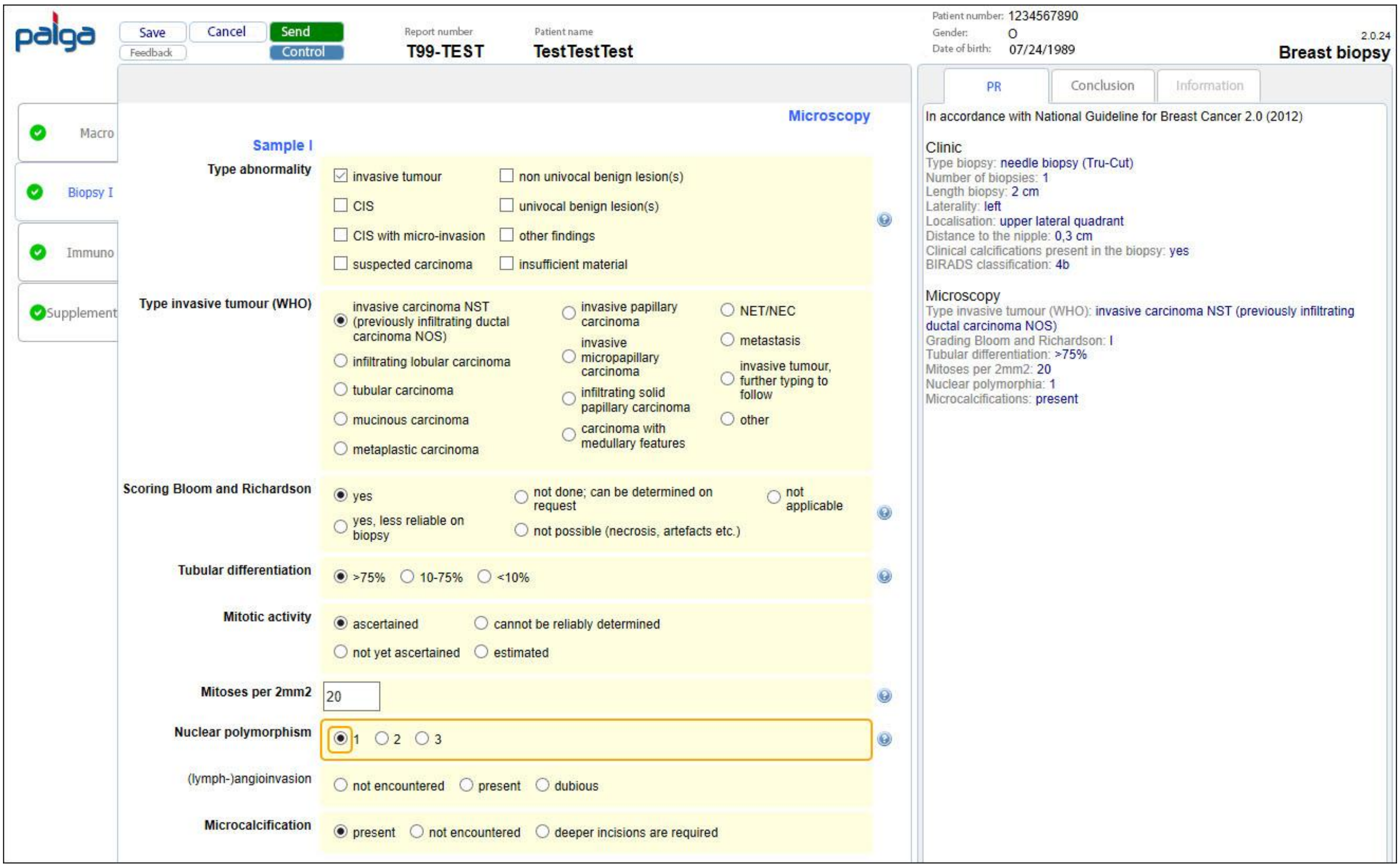

Save | Cancel | Send | Feedback | Control

Report number: T99-TEST

Patient name: TestTestTest

Patient number: 1234567890
Gender: O
Date of birth: 07/24/1989

2.0.24

Breast biopsy

Macro | Biopsy I | Immuno | Supplement

Microscopy

Sample I

Type abnormality
- [x] invasive tumour
- [ ] non univocal benign lesion(s)
- [ ] CIS
- [ ] univocal benign lesion(s)
- [ ] CIS with micro-invasion
- [ ] other findings
- [ ] suspected carcinoma
- [ ] insufficient material

Type invasive tumour (WHO)
- (•) invasive carcinoma NST (previously infiltrating ductal carcinoma NOS)
- ( ) invasive papillary carcinoma
- ( ) NET/NEC
- ( ) infiltrating lobular carcinoma
- ( ) invasive micropapillary carcinoma
- ( ) metastasis
- ( ) tubular carcinoma
- ( ) infiltrating solid papillary carcinoma
- ( ) invasive tumour, further typing to follow
- ( ) mucinous carcinoma
- ( ) carcinoma with medullary features
- ( ) other
- ( ) metaplastic carcinoma

Scoring Bloom and Richardson
- (•) yes
- ( ) not done; can be determined on request
- ( ) not applicable
- ( ) yes, less reliable on biopsy
- ( ) not possible (necrosis, artefacts etc.)

Tubular differentiation
(•) >75% ( ) 10-75% ( ) <10%

Mitotic activity
- (•) ascertained
- ( ) cannot be reliably determined
- ( ) not yet ascertained
- ( ) estimated

Mitoses per 2mm2 20

Nuclear polymorphism (•) 1 ( ) 2 ( ) 3

(lymph-)angioinvasion ( ) not encountered ( ) present ( ) dubious

Microcalcification (•) present ( ) not encountered ( ) deeper incisions are required

PR | Conclusion | Information

In accordance with National Guideline for Breast Cancer 2.0 (2012)

Clinic
Type biopsy: needle biopsy (Tru-Cut)
Number of biopsies: 1
Length biopsy: 2 cm
Laterality: left
Localisation: upper lateral quadrant
Distance to the nipple: 0,3 cm
Clinical calcifications present in the biopsy: yes
BIRADS classification: 4b

Microscopy
Type invasive tumour (WHO): invasive carcinoma NST (previously infiltrating ductal carcinoma NOS)
Grading Bloom and Richardson: I
Tubular differentiation: >75%
Mitoses per 2mm2: 20
Nuclear polymorphia: 1
Microcalcifications: present

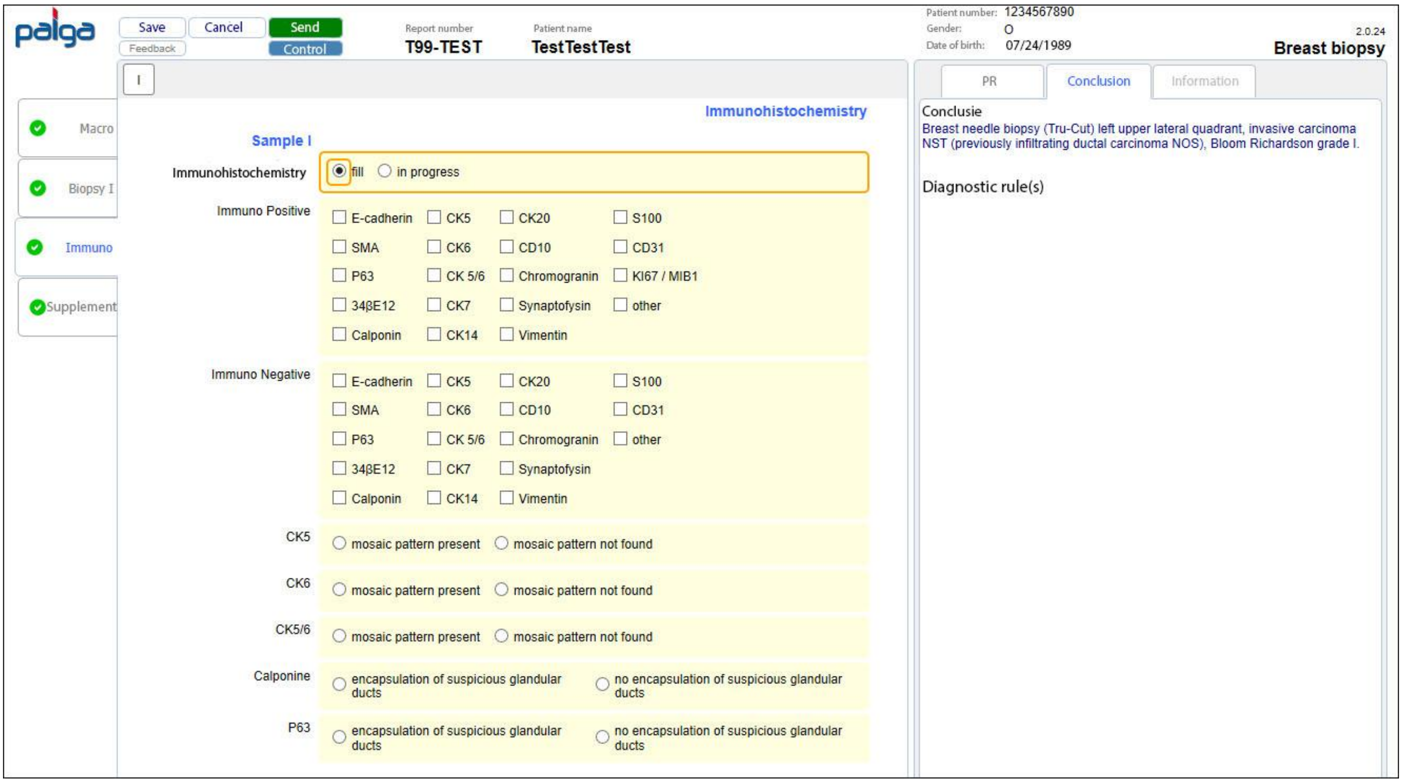

Save | Cancel | Send | Feedback | Control

Report number: T99-TEST

Patient name: TestTestTest

Patient number: 1234567890
Gender: O
Date of birth: 07/24/1989

2.0.24

Breast biopsy

I

Macro | Biopsy I | Immuno | Supplement

Immunohistochemistry

Sample I

Immunohistochemistry (•) fill ( ) in progress

Immuno Positive
- [ ] E-cadherin [ ] CK5 [ ] CK20 [ ] S100
- [ ] SMA [ ] CK6 [ ] CD10 [ ] CD31
- [ ] P63 [ ] CK 5/6 [ ] Chromogranin [ ] KI67 / MIB1
- [ ] 34βE12 [ ] CK7 [ ] Synaptofysin [ ] other
- [ ] Calponin [ ] CK14 [ ] Vimentin

Immuno Negative
- [ ] E-cadherin [ ] CK5 [ ] CK20 [ ] S100
- [ ] SMA [ ] CK6 [ ] CD10 [ ] CD31
- [ ] P63 [ ] CK 5/6 [ ] Chromogranin [ ] other
- [ ] 34βE12 [ ] CK7 [ ] Synaptofysin
- [ ] Calponin [ ] CK14 [ ] Vimentin

CK5 ( ) mosaic pattern present ( ) mosaic pattern not found

CK6 ( ) mosaic pattern present ( ) mosaic pattern not found

CK5/6 ( ) mosaic pattern present ( ) mosaic pattern not found

Calponine ( ) encapsulation of suspicious glandular ducts ( ) no encapsulation of suspicious glandular ducts

P63 ( ) encapsulation of suspicious glandular ducts ( ) no encapsulation of suspicious glandular ducts

PR | Conclusion | Information

Conclusie
Breast needle biopsy (Tru-Cut) left upper lateral quadrant, invasive carcinoma NST (previously infiltrating ductal carcinoma NOS), Bloom Richardson grade I.

Diagnostic rule(s)
